# One-Year Prevalence of Perceived Medical Errors or Near Misses and Its Association with Depressive Symptoms among Chinese Medical Professionals: A Propensity Score Matching Analysis

**DOI:** 10.3390/ijerph19063286

**Published:** 2022-03-10

**Authors:** Meixia Xu, Yifan Wang, Shuxin Yao, Rongju Shi, Long Sun

**Affiliations:** 1 Department of Current Situation and Policy, School of Marxism, Shandong Women’s University, Jinan 250300, China; xumeixia@sdwu.edu.cn (M.X.); yaoshuxin@163.com (S.Y.); shirju@163.com (R.S.); 2 Centre for Health Management and Policy Research, School of Public Health, Cheeloo College of Medicine, Shandong University, 44 Wenhuaxi Road, Jinan 250012, China; yifanw@mail.sdu.edu.cn; 3 NHC Key Laboratory of Health Economics and Policy Research (Shandong University), Jinan 250012, China

**Keywords:** medical errors or near misses, depressive symptoms, medical professional, propensity score matching, China

## Abstract

**Objective:** Medical errors or near misses (MENM) may cause serious negative outcomes for the patients. However, medical professionals with MENM may also be secondary victims. Although the association between MENM and depression among medical professionals has been explored in several previous studies, the possible causal relationship has been explored less, especially in China. In this study, our first aim was to determine the prevalence of MENM among Chinese medical professionals. We also wanted to explore the causal effect of MENM on depressive symptoms based on a propensity-score matching analysis. **Methods:** A cross-sectional study was conducted among medical professionals in Chinese public general hospitals, and 3426 medical professionals were analyzed in this study. The Center for Epidemiologic Studies Depression (CES-D) scale was used to assess depressive symptoms. Social support was measured by the Multidimensional Scale of Perceived Social Support (MSPSS). MENM, social-demographic variables, occupational characteristics, and physical disease were also evaluated in this study. **Results:** The one-year prevalence of perceived MENM was 2.9% among medical professionals in Chinese public general hospitals. The results of logistic regressions showed that working hours/week (OR = 1.02, *p* < 0.05) and depressive symptoms (OR = 1.05, *p* < 0.001) were associated with MENM. After propensity score matching, depressive symptoms were associated with MENM (OR = 1.05, *p* < 0.001) among medical professionals. The associations between occupational characteristics, physical disease, social support, and MENM were not supported by this study. **Conclusions**: The one-year prevalence of MENM was low in Chinese public general hospitals, and based on our propensity score matching analyses, the occurrence of MENM may cause depressive symptoms in medical professionals. A bigger effort by health systems and organizations may be helpful for reducing MENM.

## 1. Background

It is well known that medical professionals play very important roles in patient care and safety, and any medical errors or near misses (MENM) may result in serious negative outcomes for the patients. In the United States (US), medical errors have been the third-leading cause of death [1]. Although this ranking is not available for other countries, a high prevalence of MENM has been reported in some countries [2,3,4]. However, the prevalence of MENM has not been studied well in China, which is a country with the largest population and number of medical services in the world.

Although we know that MENM can cause serious negative outcomes for patients, we should understand that MENM is inevitable, because medical professionals are imperfect humans [5,6]. There is no doubt that patients are the primary victims of MENM. Albert Wu coined the term “second victim” to claim that doctors who made mistakes needed help, too [7]. Although the term “second victim” is incompatible with a patient’s safety and a healthcare provider’s accountability [8], we should pay attention to the impact of MENM on medical professionals.

Indeed, there are many studies that support the association between MENM and negative psychological problems, such as depression [9], burnout [10,11], mental quality of life [12], permanent emotional scarring [13], and so on [14,15,16]. All of these studies have given us important evidence for the association between MENM and psychological problems. However, how these associations occur remains an important problem for us. On one hand, as we introduced in the last paragraph, medical errors may directly cause psychological problems for medical professionals [17]. On the other hand, medical professionals with psychological problems may be at a higher risk of negligence and improper behavior when providing medical care, which may cause further medical errors [18]. As a result, the possible causal relationship between the two needs to be explored.

To address these gaps in our understanding, a cross-sectional study was conducted among medical professionals in public general hospitals in Shandong province, China. The first aim for this study was to investigate the one-year prevalence of perceived MENM among medical professionals, and the second aim was to explore the possible effect of MENM on depressive symptoms among them. Propensity score matching is an emerging matching technique for causal inference in observational research [19]. It has also been applied for eliminating the imbalance between intervention and non-intervention groups, and used in many studies worldwide in recent years [20,21]. The findings of this study will not only be helpful for understanding the prevalence of MENM in public general hospitals, but they will also provide further understanding of the causal effect of MENM on depressive symptoms based upon our propensity score matching analyses.

## 2. Methods

### 2.1. Setting and Participants

This study was conducted among medical professionals working in public general hospitals in Shandong province, China. Shandong province is in the east of China, where the size of the population and the number of medical workers are ranked second and first for all Chinese provinces, respectively [22,23]. To recruit medical professionals for this cross-sectional study, a multiple stratified random cluster sampling method was performed according to the following steps. Firstly, we divided the 17 cities in Shandong into 3 classifications according to the gross domestic product (GDP) per capita from 2018 [22], and one city was randomly selected from each classification. Secondly, one municipal hospital was randomly selected from each of the selected cities. Thirdly, three counties (districts) were randomly selected from each of the selected cities. In each of the selected counties (districts), one county-level hospital (district-level hospital) was randomly selected from the selected counties (districts). Finally, we selected 3 municipal hospitals and 9 county-level hospitals for this study. In these hospitals, we selected 3 inpatient areas from each department in the municipal hospitals, and 2 inpatient areas from each department in the county-level hospitals. Doctors, nurses, and medical technicians, who worked on the survey date were approached to participate in this study. In total, we collected 3426 valid questionnaires for this study.

### 2.2. Data Collection

The survey for this study was performed between December 2018 and January 2019. Medical professionals received the questionnaires individually. The questionnaires were filled out anonymously, and there was no reward for these medical professionals. We also asked two trained postgraduate students in the hospital to answer the questions and collect the questionnaires on the survey date.

### 2.3. Measures

#### 2.3.1. Medical Errors or Near Misses (MENM)

MENM was assessed with the question, “have you ever made any MENM in the last year?” The answers were yes (1) and no (0). Medical professionals with positive responses were considered as making a MENM in the last year. This assessment method for MENM has been used in previous studies [24,25].

#### 2.3.2. Depressive Symptoms

The Chinese version of the Center for Epidemiologic Studies Depression (CES-D) scale was used to evaluate depressive symptoms in this study [26,27]. The Chinese version of CES-D has been validated with good reliability for different populations [28,29]. It contains 20 items, such as, “I felt that everything I did was an effort”, “I felt fearful”, and so on. For each item, the scores were ranked from 0 to 3 according to the frequency of depressive symptoms in the past week. The reliability and validity of this scale has been proven in other countries around the world [30,31]. In this study, Cronbach’s alpha for CES-D was 0.852.

#### 2.3.3. Social-Demographic Variables

Gender was coded as male (1) and female (0). Age was calculated according to the medical professionals’ date of birth, which was analyzed as a continuous variable. Marital status was assessed by a choice between single, married, divorced, widowed, and others. Since there were only a few medical professionals that were divorced, widowed, or others, we recoded this status as single (1), married (2), and others (3). Education was evaluated according to the medical professionals’ academic degree. Since most of medical professionals reported either a doctor, master’s, or bachelor’s degree, we recoded education as doctor (1), master (2), bachelor degree (3), and others (4).

#### 2.3.4. Occupational Characteristics

The types of medical staff included in this study were doctor (1), nursing (2), and medical technician (3). Professional title was ranked as senior (1), vice-senior (2), intermediate (3), and junior and others (4). Manager status was evaluated with a question about the medical professionals’ administrative function in their hospitals. Working hours was determined with a question about the average working hours/week for the medical professionals, and it was analyzed as a continuous variable.

#### 2.3.5. Physical Disease

The question, “if you have been diagnosed with any physical disease?” was used to evaluate physical disease for the medical professionals. The answer was yes (1) and no (0).

#### 2.3.6. Social Support

Social support was measured by the Chinese version of the Multidimensional Scale of Perceived Social Support (MSPSS) [32]. It contains 12 items with a 7-point scale for the answers, ranging from strongly disagree (1) to strongly agree (7) for each item. The items are mainly concerned with the perceived support from the subjects’ friends and family, such as”, is there a special person with whom I can share my joys and sorrows?”, “my family really tries to help me”, and so on. A high score indicates a high level of social support. In this study, Cronbach’s alpha was 0.958 for MSPSS.

### 2.4. Statistical Analysis

In this study, IBM SPSS Statistics 24.0 (web edition) and R (version 3.2.5) were used to perform the data analyses. A Student’s T-test or a one-way ANOVA was conducted to analyze the factors associated with MENM. A logistic regression was conducted to further examine the factors associated with MENM. To explore the causal effect of MENM on depressive symptoms, propensity score matching was conducted based on a 1:3 matching ratio for medical professionals.

A logistic model was used to calculate the propensity scores with MENM as the dependent variable, and the covariates included the following 10 variables: gender, age, marital status, education level, type of medical staff, professional title, manager, physical disease, working hours/week, and social support. All of the tests were two-tailed and a *p*-value of ≤0.05 was considered as statistically significant.

## 3. Results

**Figure 1:**
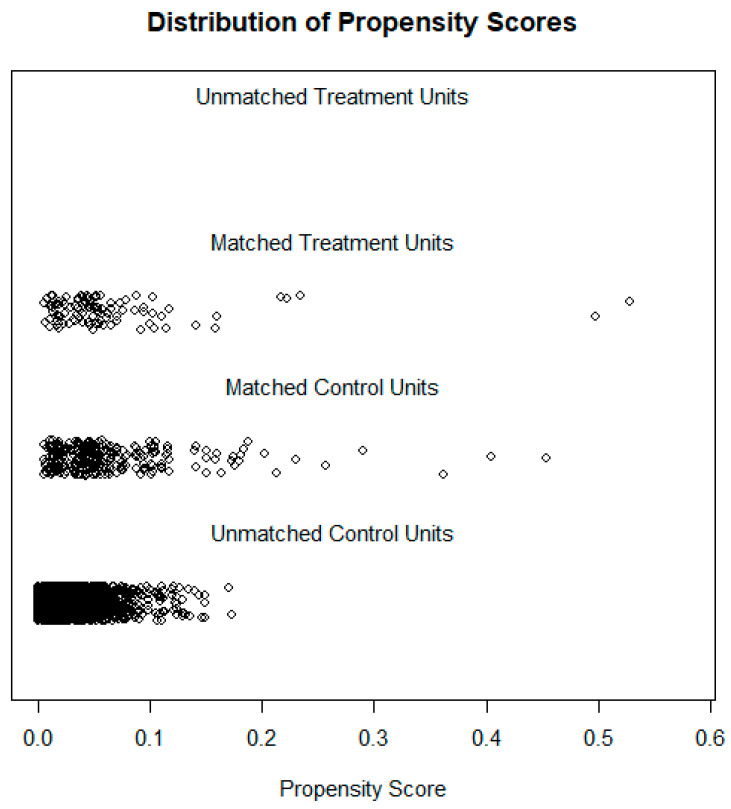
Jitter plot of the propensity score for the matched and unmatched cases.

In this study, we analyzed a total of 3426 medical professionals that worked in Chinese public general hospitals. There were 99 medical professionals (2.9%) who perceived MENM in the last year. Single analyses supported that marital status ($\chi^{2}$ = 11.84, *p* < 0.01), education level ($\chi^{2}$ = 14.73, *p* < 0.01), professional title ($\chi^{2}$ = 11.02, *p* < 0.05), working hours/week ($\chi^{2}$ = 2.74, *p* < 0.01), social support ($\chi^{2}$ = −3.70, *p* < 0.001), and depressive symptoms ($\chi^{2}$ = 6.11, *p* < 0.001) were associated with MENM. Propensity score matching was also performed to analyze the association between MENM and depressive symptoms. Figure 1 shows the jitter plot for matched and unmatched cases. Single analyses after propensity score matching were also conducted in this study. We found that all of the matched factors were not associated with MENM (*p* > 0.05), and only depressive symptoms was associated with MENM ($\chi^{2}$ = 3.92, *p* < 0.001). The detailed results for the pre-matching and post-matching are shown in Table 1.

In Table 2, logistic regressions were also performed to analyze the factors associated with MENM. The results indicated that working hours/week (OR = 1.02, *p* < 0.05), and depressive symptoms (OR = 1.05, *p* < 0.001) were associated with MENM before propensity score matching among medical professionals. After propensity score matching, depressive symptoms was also associated with MENM (OR = 1.05, *p* < 0.001) among medical professionals.

## 4. Discussion

In this study, there are several critical findings for MENM among medical professionals in Chinese public general hospitals. The first concerns the prevalence of perceived MENM, which we found affected 2.9% of medical professionals in the last year. We also found that the number of working hours per week and depressive symptoms were associated with MENM among medical professionals. The results of propensity score matching analyses further supported a causal effect of MENM on depressive symptoms. The other interesting finding was that none of the other analyzed factors were associated with MENM, with the exception of working hours per week and depressive symptoms.

For the one-year prevalence of MENM, our finding was 2.9%, which was much lower than other studies from around the world. Previous studies have reported a wide range for the prevalence of MENM. A Study among US physicians reported that the prevalence of medical errors was 10.5% in last three months [33], and another study in Poland reported that two-in-three physicians admitted to making an error in the last three months [34]. Among US medical residents, 22.5% reported committing a near miss medical error, and 6.9% reported committing a harmful medical error [35]. Among nurses, a study reported that about 40% of nurses committed a medical error during their careers [36]. All of these studies reported a higher prevalence of MENM than our results. One of the reasons for this may be due to methodological differences among these studies [37]. Another reason may be the different time frames surveyed by these studies. In our study, the time frame for perceived MENM was one year, while other studies mainly used a time frame of a lifetime. The effect of shyness on perceived MENM, which was discussed in previous studies [38], may have also contributed to these differences. The Confucian values of modesty and shyness may have reduced the prevalence of perceived MENM among Chinese medical professionals [39].

The other main aim for this study was to identify the association between MENM and depressive symptoms, and we also wanted to explore the causal effect of MENM on depressive symptoms using propensity score matching analyses. Both hypotheses were supported in this study. For the association between MENM and depressive symptoms, it has been reported by several studies for different kinds of medical professionals [14,40,41]. In this study, the results of propensity score matching confirmed a causal effect of MENM on depressive symptoms. Thus, it is important that medical professionals with MENM should pay attention to their emotional problems, and that some kind of intervention is required after the occurrence of an MENM.

In this study, a positive correlation between longer working hours per week and MENM was also observed. This association has been reported by previous studies [42,43,44], which is understandable. It is known that longer working hours are associated with burnout [45,46], which is a significant risk factor for MENM [47,48,49]. Another explanation may be that medical professionals may be fatigued by working long hours, and fatigued people are more likely to make mistakes while they are working [50]. The Institute of Medicine (IOM) recently revised the duty hours and workloads for medical residents to reduce the chances of fatigue related MENM [51].

The other interesting findings of this study concerned the association between occupational characteristics, physical disease, social support, and MENM. We found that all of these factors were not associated with MENM after we controlled for working hours per week and depressive symptoms. Although we should be cautious about concluding that these factors are not associated with MENM, previous studies have shown that most MENM are the result of system issues [52], and that MENM were related to program-specific, modifiable factors, involvement in program-related decisions, and call structure [53]. The previous studies also identified that the main factors associated with MENM were occupational stress, mental health, and physical health [24,54,55]. It was also suggested that health systems and organizations could make a bigger effort to help reduce MENM.

There were some limitations, which should be considered when interpreting the results of this study. Firstly, this is a cross-sectional study. Although propensity score matching analyses may identify causal relationships, we should be cautious about the strength of these causal relationships. Some longitudinal studies should be conducted to confirm the causal relationship between MENM and depressive symptoms. Secondly, since the prevalence of MENM, and the factors analyzed by this study, were self-evaluated by medical professionals, it may underestimate the prevalence of MENM and introduce a bias to the findings of this study. Thirdly, all participants were medical professionals working in Chinese public general hospitals, and the findings may be not relevant to other kinds of hospitals in other countries. Fourthly, only depressive symptoms were analyzed in this study, while other variables, such as health-related quality of life and quality of medical care, were not analyzed.

Considering these limitations, this study provides some critical findings and implications for understanding the prevalence of MENM and its effect on depressive symptoms. Firstly, the one-year prevalence of MENM was low in Chinese public general hospitals. Secondly, the occurrence of MENM may cause depressive symptoms in medical professionals. Thirdly, the efforts of health systems and organizations could help to reduce MENM. These findings indicate that medical professionals that have experienced MENM are at a higher risk for depressive symptoms, and they will need help, so they can be considered a “second victim”.

## Figures and Tables

**Table 1:** Description of the sample and single analysis for the factors associated with MENM among medical professionals before and after propensity score matching.

Variables	Pre-Matching				Post-Matching			
	$\bar{X}$ ± S/n(%)	MENM [n (%)]		t/χ²	$\bar{X}$ ± S/n(%)	MENM [n (%)]		t/χ²
		Yes	No			Yes	No	
Observations	3426 (100.0)	99 (2.9)	3327 (97.1)	--	396 (100.0)	99 (25.0)	297 (75.0)	--
Depressive symptoms	13.62 ± 9.98	20.97 ± 10.06	14.53 ± 10.33	6.11 ***	17.25 ± 11.11	20.97 ± 10.06	16.01 ± 11.18	3.92 ***
Gender				2.20				0.03
Male	919 (26.8)	33 (3.6)	886 (96.4)		129 (32.6)	33 (25.6)	96 (74.4)	
Female	2507 (73.2)	66 (2.6)	2441 (97.4)		267 (67.4)	66 (24.7)	201 (75.3)	
Age	35.14 ± 8.42	35.16 ± 9.04	35.14 ± 8.40	0.02	34.99 ± 9.14	35.16 ± 9.04	34.94 ± 9.18	0.21
Married status				11.84 **				0.02
Single	577 (16.8)	29 (5.0)	548 (95.0)		114 (28.8)	29 (25.4)	85 (74.6)	
Married	2802 (81.8)	68 (2.4)	2734 (97.6)		274 (69.2)	68 (24.8)	206 (75.2)	
Others	47 (1.4)	2 (4.3)	45 (95.7)		8 (2.0)	2 (25.0)	6 (75.0)	
Education				14.73 **				0.24
Doctor	56 (1.6)	6 (10.7)	50 (89.3)		23 (5.8)	6 (26.1)	17 (73.9)	
Master	562 (16.4)	10 (1.8)	552 (98.2)		37 (9.3)	10 (27.0)	27 (73.0)	
Bachelor	2368 (69.1)	70 (3.0)	2298 (97.0)		279 (70.5)	70 (25.1)	209 (74.9)	
Others	440 (12.8)	13 (3.0)	427 (97.0)		57 (14.4)	13 (22.8)	44 (77.2)	
Types of medical professionals				0.37				0.29
Doctor	1268 (37.0)	39 (3.1)	1229 (96.9)		153 (38.6)	39 (25.5)	114 (74.5)	
Nursing	1695 (49.5)	46 (2.7)	1649 (97.3)		192 (48.5)	46 (24.0)	146 (76.0)	
Medical technician	463 (13.5)	14 (3.0)	449 (97.0)		51 (12.9)	14 (27.5)	37 (72.5)	
Professional title				11.02 *				0.12
Senior	109 (3.2)	7 (6.4)	102 (93.6)		26 (6.6)	7 (26.9)	19 (73.1)	
Vice-senior	303 (8.8)	14 (4.6)	289 (95.4)		56 (14.1)	14 (25.0)	42 (75.0)	
Intermediate	1170 (34.2)	24 (2.1)	1146 (97.9)		93 (23.5)	24 (25.8)	69 (74.2)	
Junior and others	1844 (53.8)	54 (2.9)	1790 (97.1)		221 (55.8)	54 (24.4)	167 (75.6)	
Manager				0.26				0.01
Yes	659 (19.2)	21 (3.2)	638 (96.8)		83 (21.0)	21 (25.3)	62 (74.7)	
No	2767 (80.8)	78 (2.8)	2689 (97.2)		313 (79.0)	78 (24.9)	235 (75.1)	
Working hours/week	47.25 ± 9.27	50.26 ± 10.11	47.61 ± 9.45	2.74 **	50.20 ± 10.18	50.26 ± 10.11	50.18 ± 10.22	0.07
Physical disease				3.02				0.49
Yes	457 (13.3)	19 (4.2)	438 (95.8)		67 (16.9)	19 (28.4)	48 (71.6)	
No	2969 (86.7)	80 (2.7)	2889 (97.3)		329 (83.1)	80 (24.3)	249 (75.7)	
Social support		57.40 ± 15.51	62.61 ± 13.74	−3.70 ***	58.09 ± 15.14	57.40 ± 15.51	58.31 ± 15.03	−0.52

Note: ***: *p* < 0.001; **: *p* < 0.01; *: *p* < 0.05.

**Table 2:** Logistic regressions for the association between depressive symptoms and MENM among medical professionals before and after propensity score matching [OR (95% CI)].

Variables	Pre-Matching	Post-Matching
Depressive symptoms	1.05 (1.03, 1.07) ***	1.05 (1.02, 1.07) ***
Male	1.13 (0.67, 1.89)	1.00 (0.55, 1.81)
Age	1.00 (0.96, 1.04)	1.01 (0.96, 1.06)
Married Status (Ref. = Others)		
Single	1.29 (0.28, 6.03)	1.02 (0.17, 6.12)
Married	0.63 (0.14, 2.78)	0.93 (0.17, 5.10)
Education (Ref. = Others)		
Doctor	1.70 (0.51, 5.66)	1.27 (0.33, 4.83)
Master	0.50 (0.20, 1.26)	1.32 (0.45, 3.90)
Bachelor	1.02 (0.54, 1.91)	1.16 (0.56, 2.39)
Types of medical professionals (Ref. = Medical technician)		
Doctor	0.87 (0.44, 1.72)	1.08 (0.49, 2.40)
Nursing	0.96 (0.49, 1.84)	1.01 (0.47, 2.18)
Professional title (Ref. = Junior and others)		
Senior	3.02 (0.86, 10.60)	0.91 (0.21, 3.89)
Vice-senior	2.38 (0.91, 6.18)	0.79 (0.25, 2.51)
Intermediate	0.93 (0.51, 1.72)	0.97 (0.47, 1.99)
Manager	0.81 (0.41, 1.59)	1.00 (0.45, 2.23)
Working hours/week	1.02 (1.00, 1.04) *	1.00 (0.97, 1.02)
Physical disease	1.11 (0.64, 1.94)	1.01 (0.53, 1.89)
Social support	0.99 (0.98, 1.01)	1.01 (0.99, 1.03)
Constant	0.01 ***	0.07
R^2^	0.08	0.06

Note: ***: *p* < 0.001; *: *p* < 0.05. CI denotes to confidence interval.

## Data Availability

The datasets used and/or analyzed during the current study are available from the corresponding author on reasonable request.
